# Hydrolytic stability of anticancer drugs and one metabolite in the aquatic environment

**DOI:** 10.1007/s11356-021-14360-0

**Published:** 2021-06-08

**Authors:** Michał Toński, Joanna Dołżonek, Piotr Stepnowski, Anna Białk-Bielińska

**Affiliations:** Department of Environmental Analysis, Faculty of Chemistry, University of Gdańsk, ul. Wita Stwosza 63, 80-308 Gdańsk, Poland

**Keywords:** Anticancer drugs, Environment, Hydrolysis, Metabolite, Degradation, Stability

## Abstract

Due to the genotoxic, carcinogenic and teratogenic mechanism of action, anticancer drugs are highly hazardous compounds. Their occurrence, fate, and effects in the environment have not been systematically studied as compared to other medicaments. Therefore, reliable data, including their stability and persistency, is required in order to assess it. Taking into account, that hydrolysis is one of the most important factors regarding stability of chemicals in water, the aim of our study was to investigate the hydrolytic stability of five commonly used anticancer drugs (ifosfamide, cyclophosphamide, 5-fluorouracil, imatinib, and methotrexate) and one metabolite (7-hydroxymethotrexate), as the systematized and coherent data available is limited. The hydrolysis studies have been prepared according to the OECD 111 procedure to obtain standardized and comparable results. The preliminary tests at pH 4, 7, and 9 and 50 °C show that only cyclophosphamide and ifosfamide are unstable, whereas the estimated t_1/2_ at 25 °C is >1 year for other investigated compounds. Moreover, much more detailed experiments were performed and indicate that at environmentally relevant temperatures, cyclophosphamide, and ifosfamide would be quite persistent in the terms of hydrolytic stability. Moreover, the preliminary investigation on the hydrolysis products was performed.

**Supplementary Information:**

The online version contains supplementary material available at 10.1007/s11356-021-14360-0.

## Introduction

Anticancer drugs belong to the group of pharmaceuticals whose presence in the environment has gain the scientific attention much later than other popular groups of medicaments, such as nonsteroidal anti-inflammatory drugs (NSAIDs) or antimicrobials. However, due to their genotoxic, mutagenic, and teratogenic mechanism of action, they belong to highly hazardous compounds (Mišík et al. 2014). Their occurrence, fate, and effects in the environment have not been systematically studied as compared to other pharmaceuticals, even those used in smaller quantities. Nevertheless, the available data is quite diversified in the terms of the threat and risk they might pose. On one hand, their concentrations in hospital effluents (up to hundreds μg L^−1^ (Cristóvão et al. 2019; Santana-Viera et al. 2020)) are alarmingly high as well as their presence in wastewater effluents (Hilton and Thomas 2003; Olalla et al. 2018; Santana-Viera et al. 2019) is concerning. On the other hand, the concentrations in natural waters are on the level of ng L^−1^ (Azuma 2018; Ferrando-Climent et al. 2014; Kosjek and Heath 2011), if the anticancer drugs are detected at all (Santos et al. 2018; Ternes 1998). In some cases, even concentrations in wastewaters are on the level of ng L^−1^ (Negreira et al. 2013a). Additionally, the available ecotoxicological data shows that even though some of the cytostatics are highly toxic to some tested organisms (Białk-Bielińska et al. 2017), in most cases the concentrations that cause toxic effect are still much higher than those confirmed or predicted in the environment (Martín et al. 2014). Nevertheless, some authors indicate that the problem is more complex than it was assumed in many earlier publications. They highlight the fact that toxic (cytotoxic) effects would be different for adults than children, pregnant women and their fetuses, or elders (Kümmerer 2009). Researchers raise also the topic of mixtures of the pollutants (Parrella et al. 2014a). Single compound may be less or more toxic than in a mixture with others, which is quite challenging to measure (Česen et al. 2016; da Fonseca et al. 2019). Nevertheless, it is also suspected that the presence of cytostatic residues in the environment may lead to systemic environmental effects that in the worst case could lead to extinction of susceptible organisms, while in exposed humans to increased cancer incidence or reproductive defects (https://cordis.europa.eu/project/id/265264, Isidori et al. 2016; Parrella et al. 2014b).

Another matter that should be considered is that some of those pharmaceuticals are only prodrugs; thus, the threat may also come from the active compounds created during metabolism or after excretion (Kosjek et al. 2013; Vredenburg et al. 2014). This problem is also associated with an issue of metabolites and degradation products (also called transformation products), which are produced during the metabolic changes or under environmental conditions. If native compounds are detected in wastewaters (in the concentrations range of ng L^−1^ up to μg L^−1^) and only some part of the applied drugs are excreted in unchanged forms, it becomes obvious that more attention should be paid to those transformation products. Especially if they pose a risk of being stable, toxic, biologically active, or may be transformed back to the original pharmaceutical and are in similar or higher concentrations than the actual drug. For example, cyclophosphamide is oxidized into 4-hydroxycyclophosphamide, which reaches the cells and undergoes another changes, which result in the formation of acrolein and phosphoramide mustard (Brummaier et al. 2013). 5-fluorouracil may be administered as a pharmaceutical itself; however, it is also a product of prodrugs: capecitabine or tegafur transformation (Azuma 2018; Franquet-Griell et al. 2015).

Therefore, reliable data is required to assess the potential risk posed by these pharmaceuticals as well as their transformation products in the environment. One of the fundamental issues of their presence is the persistency in the aquatic environment, which is expressed, among other factors, in hydrolytic stability. Some authors have tested anticancer drugs in terms of stability in water. However, the studies were designed and conducted in different ways, so unified data are not available. For example, mitoxantrone dissolved in hard water was completely transformed into four stable transformation products, while 81 % of chlorambucil was transformed at the beginning of the test and remaining 19 % degraded within 24 h when the initial concentration was 10 ng μL^−1^; however, there was an instant and complete transformation at 2 ng μL^−1^ (Gómez-Canela et al. 2015). Other study shows that the group of anticancer drugs is diversified in terms of its hydrolytic stability. It was discovered that seven out of 16 investigated analytes were highly unstable in milli-Q water, namely daunorubicin, doxorubicin, vinblastine, chlorambucil, vincristine, irinotecan, and melphalan.(Franquet-Griell et al. 2017). However, the rest of the analytes (cyclophosphamide, ifosfamide, mycophenolic acid, prednisone, cytarabine, gemcitabine, capecitabine, etoposide, and megestrol) were described as stable. The experiments mentioned above were conducted in mild conditions, such as room temperature and natural pH of the water that was used. Moreover, there have been also some papers published, which main goal was to trigger hydrolysis in different conditions (usually low and high values of pH and temperature). For example, methotrexate degradation was investigated between pH 8.5 to 12 and at the temperature even up to 85 °C (Chatterji and Gallelli 1978). Cyclophosphamide and ifosfamide were tested under very low and high pH and temperature 20–70 °C (Gilard et al. 1994, 1999; Muñoz et al. 1996). Other papers refer directly to stress conditions in order to observe degradation and formation of transformation products, like in case of imatinib (Alkharfy et al. 2013; Szczepek et al. 2007) or 5-fluorouracil (Yadav et al. 2012). These examples show, that even though there is soma data regarding the hydrolysis of anticancer drugs available, the variety of the applied methods and conditions (including pH, temperature, matrix composition, duration of the test, neglecting the elimination of other factors—like using autoclaved water or elimination of air from the vessels), leads to the difficulties in comparing obtained results and sometimes even extrapolate them to environmental conditions. Therefore, there still is a necessity to investigate this topic in more ordered and arranged manner, especially in the environmental context.

**Fig. 1:**
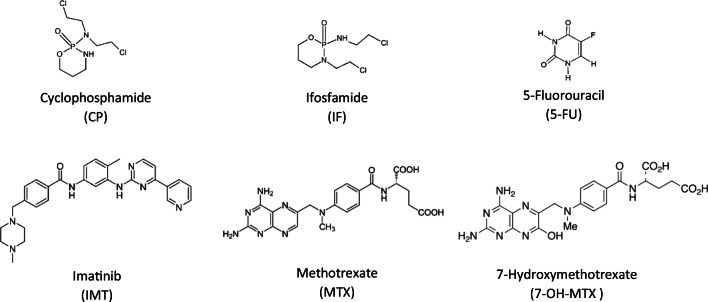
The structures of the analytes included in this research

For this reasons, the aim of our study was to investigate the stability of five selected anticancer drugs (Fig. 1): cyclophosphamide, ifosfamide, 5-fluorouracil, methotrexate, imatinib, and one metabolite (7-hydroxymethotrexate) in the aquatic environment in terms of their susceptibility to hydrolysis, as there is limited information available on this topic. These analytes were chosen as representatives of different types of anticancer drugs, such as alkylating agents, antimetabolites, or kinase inhibitors. Moreover, they have been introduced to medicine at different times, from the oldest cyclophosphamide and ifosfamide to the newest imatinib. They are also one of the most popular anticancer drugs (Gómez-Canela et al. 2017; Lin and Lin 2014; Steger-Hartmann et al. 1996), which may also be harmful according the toxicity to selected model organisms, like 5-FU to algae or methotrexate to duckweed (Białk-Bielińska et al. 2017); thus, the data on their stability is essential. Taking into account that hydrolysis is one of the most crucial processes in the ecosystem that may lead to the elimination of toxic compounds; it is of utmost importance to fill this gap in the knowledge. The hydrolysis studies have been performed according to the standardized OECD 111 procedure in order to fulfill the basic requirements for the ERA of pharmaceuticals as well as to be able to compare the obtained results between different pharmaceuticals or results from other research groups. As such, degradation processes may also lead to the formation of new compounds (degradation products); the preliminary investigations if there are any degradation products present in the samples after the tests have been conducted.

## Materials and methods

### Materials

The analytes used in this study: cyclophosphamide monohydrate (CAS: 6055-19-2, Sigma Aldrich), ifosfamide (CAS: 2778-73-2, Sigma-Aldrich), 5-fluorouracil (CAS: 51-21-8, Sigma-Aldrich), imatinib mesylate (CAS: 220127-57-1, Santa Cruz Biotechnology), methotrexate (CAS: 59-05-2, Sigma-Aldrich), 7-hydroxymethotrexate (CAS: 5939-37-7, Toronto Research Chemicals, Canada). The stock solutions (1000 mg L^−1^) of cyclophosphamide, ifosfamide, 5-fluorouracil, and imatinib were prepared in methanol and kept in the freezer (−18 °C), whereas stock solutions of methotrexate and 7-hydroxymethotrexate (500 mg L^−1^) were prepared in DMSO and kept in the cooler (4 °C). The working solutions of the analytes were prepared in specific buffers in concentration of 10 mg L^−1^ each. Even though such concentration is higher than it is found in the environment, if the hydrolysis process follows a pseudo-first-order reaction the half-lives are independent of concentration (OECD 2004). Moreover, the OECD 111 guideline permits using concentrations up to 10^−2^–10^−3^ M. In our study, the applied concentrations (10 mg L^−1^) of cyclophosphamide and ifosfamide correspond to around 3.83 × 10^−5^ M, which is much below the permitted threshold.

### Hydrolysis experiments

**Fig. 2:**
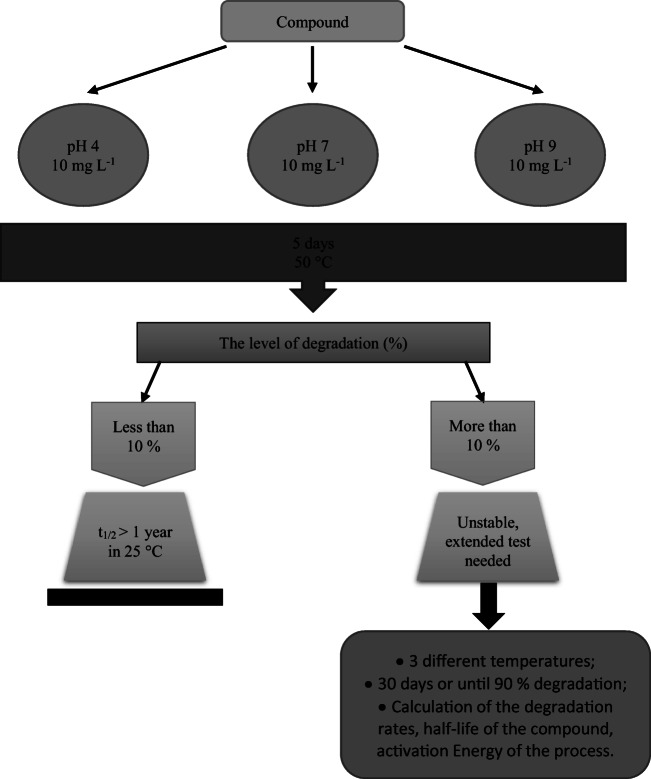
The outline of the experiments conducted in this study

The hydrolysis experiments were performed according to the OECD 111 procedure, therefore all details of the experiments can be found there (OECD 2004) or in our previous papers (Białk-Bielińska et al. 2012; Maszkowska et al. 2014; Toński et al. 2019). Briefly, the OECD Guideline 111 (OECD 2004), requires a preliminary test and if the compound degrades in more than 10 %, extended test (Fig. 2). The incubation was performed in the dark, in order to avoid the influence of the photolysis on degradation of the analytes. The water used for the preparation of buffers was autoclaved, all glassware was clean, flushed with methanol, and dried in high temperature. Samples were closed in glass tubes with a sealed cap. The air from above the solution was removed by a nitrogen stream just before capping. In the preliminary test, solutions of single analytes were prepared in each tested pH: 4, 7, and 9, which is required by the OECD guidelines. There was also a blank sample prepared for each pH. Samples in the preliminary test were prepared in two parallel replicates. Moreover, the complete preliminary test was repeated twice. The preliminary test was performed at 50 °C for 5 days. Quite high temperature of the test accelerates the degradation process and helps to separate the most stable compounds from those susceptible to hydrolysis. Hence, the analytes that degraded more than 10 % in the preliminary test were than subjected to the extended test. In the case of the extended tests, 7 measuring points were provided during the time of the test. Each measuring point was performed in two parallel replicates. The analytes determined in preliminary test as unstable at the certain pH were incubated at three different temperatures: 20, 50, and 70 °C, as required by the OECD guidelines (one temperature below 25 °C, required 50 °C and a temperature of maximum 70 °C). The test was conducted for 30 days or to 90 % elimination of a compound. Obtained results were used to determine the hydrolysis rate constant (k) using equation:

1 $$ \mathrm{l}n\frac{C_t}{C_0}= kt $$

where *t*, time; *c*_*t*_, *c*_*0*,_ concentration of the analyte at a time (0 means the beginning of the test)*; k*, rate constant.

Based on this, the t_1/2_ was determined by using the equation:

2 $$ {t}_{\frac{1}{2}}=\frac{\ln 2}{k} $$

where *t*_*1/2*_, half-life of the analyte; *k*, rate constant.

By obtaining at least two rate constants for two different temperatures, it is possible to calculate the rate constants and, in consequence, half-lives at other temperatures, according to the Arrhenius equation:

3 $$ k=A\times {e}^{\frac{-E}{R\times T}}\ \mathrm{or}\ \ln k=\frac{-E}{R\times T}+\ln A $$

Therefore, these equations were applied to extrapolate the obtained results and calculate the rate constants and half-lives of unstable anticancer drugs in more environmentally relevant temperatures (4, 10, or 15 °C), which are typical to natural waters.

### HPLC-UV/Vis and LC-MS/MS analysis

The HPLC-UV/Vis technique was applied to determine the level of hydrolytic degradation of the analytes. In the preliminary test, the control and blank samples were analyzed at the beginning and the end of the test. Based on the obtained results, the percentage of degradation of specific compound was calculated. In the extended test, the samples collected in the time intervals were analyzed, and peak areas were compared to those obtained at the beginning of the test. This was used to determine the kinetic of this hydrolysis process by using the Equation 1. The conditions of the analysis for every compound are presented in the **Table S1**. In general, each analyte was analyzed by a simple HPLC method with UV/Vis detection. Gemini C18 (Phenomenex, USA), 5 μm, 110 Å, 150 mm × 4.6 mm chromatographic column was applied for each method and three different mobile phases were used (water or 0.1 % HCOOH) mixed with the acetonitrile. The conditions applied during the LC-MS analysis with an ion trap detector for the qualitative analysis of the samples collected after the extended tests at 20 and 70 °C are presented in the **Table S2**. Control, blank, and test samples were analyzed with the LC-MS system in order to evaluate if any transformation products of investigated compounds are present in these samples.

## Results and discussion

### Preliminary test

**Fig. 3:**
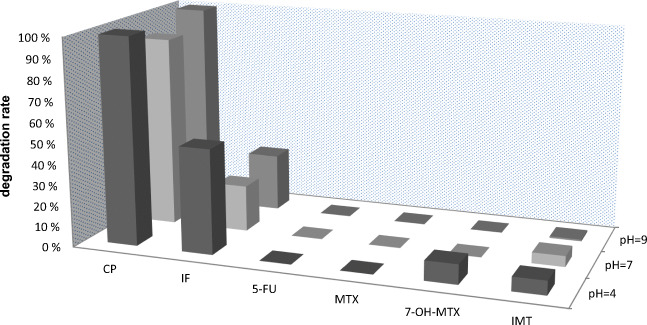
Degradation level of the investigated compounds during their incubation at 50 °C for 5 days at three different pH values in the preliminary test

The main purpose of this test is to screen and separate compounds susceptible to hydrolysis from those hydrolytically stable. It gives simple and efficient information, which is crucial in comparison with other pharmaceuticals/pollutants. The obtained results for the investigated cytostatics are presented in Fig. 3.

The results indicate that four (5-FU, MTX, 7-OH-MTX, IMT) out of six investigated compounds were hydrolytically stable (exemplary chromatogram obtained for 5-FU at pH 7 before and after the test can be seen as **Figure S1** in Supplementary Material). According to the OECD guideline, it can be assumed that their half-lives are at least 1 year at the temperature of 25 °C. The metabolite of methotrexate (7-hydroxymethotrexate) was slightly less stable than its parent compound in pH 4 (7 % degradation of 7-OH-MTX and 0 % of MTX); nevertheless, it was still below the 10 % threshold.

However, it was observed that cyclophosphamide and ifosfamide were susceptible to hydrolysis. CP was completely eliminated from the solution at the pH 4 and 9, and in 91.6 % at pH 7. IF (although structurally very similar to CP) was not that vulnerable to hydrolysis. The highest degradation level was observed at the pH 4 (50.4 %), while at the pH 7 and 9 the degradation was about half less (22.3 and 27.5 %, respectively). For these reasons, accordingly to the requirements of OECD 111 guideline, CP and IF at pH 4, 7, and 9 were advanced to the extended tests.

### Extended tests

**Fig. 4:**
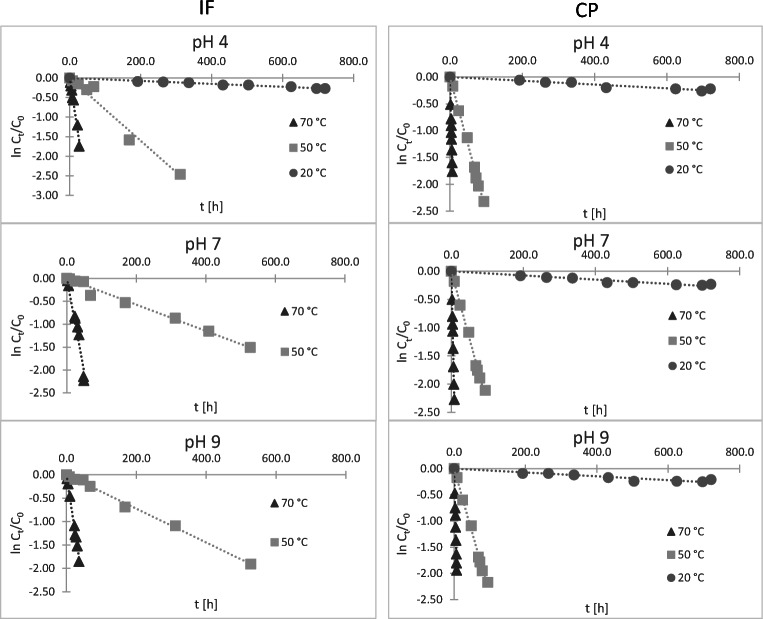
The plot of ln c_t_/c_0_ versus time for IF (on the left) and CP (on the right). There is no data for IF at pH 7 and pH 9 at 20 °C, because no degradation occurred

For complete evaluation of hydrolytic instability of CP and IF at three different pH values, their solutions were incubated at three different temperatures: 20, 50, and 70 °C. The degradation was monitored for 30 days or until 90 % of a compound was degraded. Based on the obtained results (Fig. 4) the constant rates (k) and half-lives (t_1/2_) have been calculated (Table 1).

**Table 1:** The results calculated after conducting the extended tests, where k is rate constant of the process, t_½_ is half-life of a compound, *R*^2^ is coefficient of determination and E_a_ is the activation energy of the process

Compound		CP			IF		
pH		4	7	9	4	7	9
20 °C	k [h^−1^]	3.5 × 10^−4^	3.8 × 10^−4^	3.7 × 10^−4^	3.8 × 10^−4^	n.d.	n.d.
	t_½_ [h]	1963.2	1867.9	1893.4	1843.1	n.d.	n.d.
	R^2^	0.932	0.940	0.889	0.984	n.d.	n.d.
50 °C	k [h^−1^]	2.5 × 10^−2^	2.4 × 10^−2^	2.4 × 10^−2^	8.0 × 10^−3^	2.9 × 10^−3^	3.6 × 10^−3^
	t_½_ [h]	27.6	29.4	28.8	86.5	241.9	191.7
	R^2^	0.997	0.995	0.995	0.969	0.984	0.996
70 °C	k [h^−1^]	2.7 × 10^−1^	2.8 × 10^-1^	2.7 × 10^−1^	5.9 × 10^−2^	4.1 × 10^−2^	4.9 × 10^−2^
	t_½_ [h]	2.6	2.5	2.5	11.7	16.9	14.0
	R^2^	0.998	0.997	0.995	0.983	0.965	0.996
E_a_ [kJ mol^−1^]		111.2	110.6	110.5	84.2	122.2	120.1

*n.d.*, not determined (due to no degradation in these conditions)

**Fig. 5:**
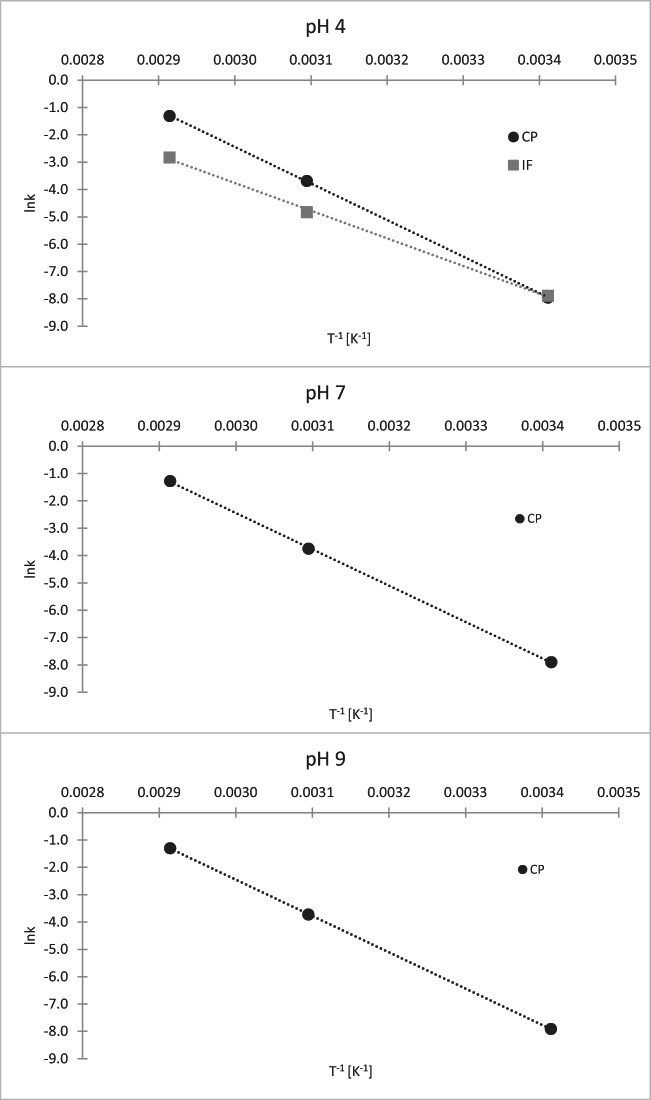
The plot of ln k_obs_ and T^−1^ for CP and IF for activation energy determination

After determining the hydrolysis rate constants it was possible to calculate activation energy (E_a_) of this process at each pH, either from plotting ln k from T^−1^ (Figure 5) when there were three k values obtained for each compound and pH (CP at pH 4, 7, and 9 as well as IF at pH 4) or from the following equation (for IF at pH 7 and 9, where only two k values were obtained) (Table 1):

4 $$ \mathrm{E}=\mathrm{R}\ \frac{\ln {\mathrm{k}}_2-\ln\ {\mathrm{k}}_1}{\left(\frac{1}{\overline{{\mathrm{T}}_1}}-\frac{1}{{\mathrm{T}}_2}\right)} $$

where R, gas constant (8.314 J/mol × K); k_1_, k_2_, rate constants for different temperatures; T, absolute temperature (K).

It was observed that the temperature has unquestionable influence on the degradation rate. In every case k increased rapidly with the increase of the temperature. At 20 °C, only around 20 % of initial amount of CP was degraded in 30 days. Moreover, IF at that temperature was unstable only at pH 4 (over 20 % of degradation in 30 days), while at pH 7 and 9 no changes in the initial concentration were observed. It shows that despite of their instability noticed in the preliminary test, at the temperature close to the environmental conditions their half-lives are high (around 80 days for CP at all pH and for IF at pH 4). Furthermore, IF at pH 9 and especially 7, which is the closest to the pH of natural waters, did not undergo hydrolysis at all (at the temperature of 20 °C). It may suggest that these compounds would be rather stable and resistant in the terms of hydrolytic stability under the average environmental conditions.

At higher temperatures, the tendency observed in the preliminary test has been confirmed. CP at each pH presents similar degradation rates, which indicates that pH has no influence on its hydrolysis rates. This is also supported with the determined values of activation energy (110.5–111.2 kJ mol^−1^). However, in the case of IF the degradation appears to be much faster at acidic conditions (t_½_ = 86.5 h at pH 4, 50 °C) than alkaline (t_½_ = 191.7 h at pH 9, 50 °C), whereas at the neutral pH it was the most stable (t_½_ = 241.9 h at pH 7, 50 °C). The activation energy values support these results; E_a_ for IF at pH 4 is distinctly lower (84.2 kJ mol^−1^) than at pH 7 and 9 (120.2 and 122.2 kJ mol^−1^ respectively).

Furthermore, to fully illustrate the degradation of CP and IF at different temperatures and pH the graphs showing degradation levels [%] in time are presented in the **Figure S2** in the Supplementary material.

Additionally, these observations confirm that CP is much more vulnerable for hydrolysis than IF, which is interesting how two isomers (like CP and IF) may differently undergo hydrolysis process. The obtained results show that differences in the temperatures may significantly change the rates of degradation. If the conclusions were drawn only based on the results from the preliminary experiments (incubation at 50 °C for 5 days), it would be assumed that both compounds, especially CP, are very unstable. Nevertheless, the extended tests showed that at the temperature closer to environmentally relevant values the degradation rates are quite low and the degradation process is notably restrained. Moreover, using Equation 3, it was possible to extrapolate the results to determine the rate constants and half-lives at the temperatures 4, 10, and 15 °C, which are ordinary for environmental waters (Table 2). The results confirm what was described earlier and show that CP and IF may be hydrolytically stable for months or even years at those temperatures.

**Table 2:** The extrapolated results of rate constants and half-lives for CP and IF at environmentally relevant temperatures

Compound		CP			IF		
pH		4	7	9	4	7	9
15 °C	k [h^−1^]	1.61 × 10^−4^	1.66 × 10^−4^	1.66 × 10^−4^	1.99 × 10^−4^	0.11 × 10^−4^	0.16 × 10^−4^
	t_½_ [days]	179	174	174	146	2578	1859
10 °C	k [h^−1^]	0.71 × 10^−4^	0.74 × 10^−4^	0.73 × 10^−4^	1.07 × 10^−4^	0.05 × 10^−4^	0.06 × 10^−4^
	t_½_ [days]	406	393	394	271	6364	4521
4 °C	k [h^−1^]	0.51 × 10^−4^	0.53 × 10^−4^	0.53 × 10^−4^	0.83 × 10^−4^	0.03 × 10^−4^	0.04 × 10^−4^
	t_½_ [days]	568	549	550	349	9219	6509

Due to a lack of data obtained with the same methodology it is difficult to compare the results presented by other authors. Nevertheless, some stability tests for cytostatics have been previously performed. CP and IF were found stable during a 48-h test in pure water at room temperature, with degradation constants 0.004 [min^−1^] (Franquet-Griell et al. 2017). Buerge et al. (2006) have observed slow degradation of CP in lake water in dark conditions and temperature of 20 °C, which resulted in a half-life of around 80 days, whereas they did not notice any IF degradation, which is in agreement with our results. Similar stability was observed by Gilard et al. (1994) at 20 °C and pH in a range 3.4–8.6, where over 95 % of initial concentration of CP was present after 7 days. Moreover, even after 17 days maximum degradation of CP was 20 %. IF was described as quite stable at the pH 6.8 and 5.5, where most of the compound was intact after a month (Gilard et al. 1999). These results were also supported with the observations made by Muñoz et al. (1996), who suggested that the degradation of IF is pH-dependent, where faster degradation occurs in acidic or alkaline conditions than at neutral pH. Moreover, at 20 °C and pH 5, a half-life of IF was around 620 days. Additionally, Booker et al. (2014) confirmed the stability of CP and IF with the application of SPARC utility, which is a chemical calculator provided by ARChem. Basing on the molecular structure of a compound, it was possible to assess that both CP and IF are stable at pH 7 and 8.1 for 5 days. Even though the authors underline that this is not an accurate tool, it can be used as a supplementary data to our results.

Various data regarding all compounds included in our study have been presented by Negreira et al. (2013a), who have tested the stability of cytostatics preliminarily at three different temperatures (4, 15, and 25 °C) for 24 h and then at 4 °C for a longer period of time. All samples were prepared in HPLC water with 4 % DMSO. They discovered that at 4 °C during 24 h test IF, CP, 5-FU, MTX, and IMT were stable, while only 86 % of 7-OH-MTX remained intact. At 15 °C and 24 h IF, CP, IMT, and 5-FU were still stable, but more than 50 % of MTX disappeared, while there was none of the 7-OH-MTX left in the solution. Degradation level of MTX increased even more (65 %) with the temperature (25 °C), while there was full degradation of 7-OH-MTX observed. Moreover, there was 11 % loss of 5-FU and 23 % of IMT at 25 °C and 24 h incubation time. However, CP and IF remained stable. Additionally, the experiment conducted at 4 °C for a more significant period of time provided information that after 9 days the concentration of MTX, 7-OH-MTX, and IMT decreased to around 50 %, while CP, IF, and 5-FU were stable for 1 month. Comparing the data described above with our results, it can be assumed that the stability of CP and IF observed is similar. However, in the case of other compounds, there are some noticeable differences. It could be due to the differences in conditions of the performed tests as the authors conducted the experiments in HPLC water, but did not present any preservation method of the water and containers during the experiments. This could lead to the biodegradation or other transformations of the analytes including the influence of the interferents from the air or the adsorption of the analytes onto the walls of the applied containers. In our study, the water was sterilized, as well as the glass containers were used, which were capped after removing the air from the above of the solution surface under the stream of nitrogen. However, Negreira et al. (2013b) also proved that acidification of the samples and content of organic modifier (such as 50 % methanol or DMSO) made 7-OH-MTX and MTX stable at previously tested conditions. It was observed that water has tremendous influence on the stability of these compounds (the more water the higher hydrolytic degradation), but acidification and high concentration of DMSO or methanol could lead to disinfection of a sample, thus prevention of biodegradation.

However, our results obtained for MTX, 7-OH-MTX, 5-FU, and IMT are in agreement with the observations of other authors. For example, the stability of MTX for at least 24 h was proved by McElnay et al. (1988) and Vrignaud et al. (2015). Additionally, in our previous study (Białk-Bielińska et al. 2017), it was observed that MTX and 7-OH-MTX under the conditions of performed ecotoxicological test were hydrolytically stable, however susceptible to photolysis. 5-FU was also found stable in the aqueous solutions for the medication purposes (Legay et al. 2014) as well as at the temperature up to 275 °C and under influence of several stress conditions (Yadav et al. 2012). Also, IMT, according to Alkharfy et al. (2013), is very stable under various conditions. Only at the neutral pH, some loss of the initial concentration (35–40 %) was observed; however, there is no information on incubation time or the temperature. It is in contrary to Szczepek et al. (2007), who claimed, that the highest stability was observed at neutral pH, while some degradation products were detected in alkaline and acidic conditions.

Nevertheless, these observations and speculations only prove that standardized methodology is necessary for comparison of the results. It is especially difficult to rely on the data provided for a different purpose than the actual evaluation of stability, where the main goal was to determine the degradation products, so the condition of the experiment were often extreme, just to observe fast degradation of a compound.

However, it can be assumed that investigated compounds are quite stable in the aquatic environment in the terms of hydrolysis. Moreover, as the data on the stability of transformation products of pharmaceuticals (like 7-OH-MTX) is very scarce, including them in this kind of research is a novel approach, which is recommended in the future studies.

### The application of LC-MS technique for the identification of potential hydrolysis products

The number of studies concerning identification of the potential hydrolytic degradation products of anticancer drugs is even more limited than those evaluating their hydrolytic stability. Taking into account papers cited in previous section, even though some authors have provided the data on stability of cytostatics, Gilard et al. (1999, 1994) and Szczepek et al. (2007) focused mainly on determining the degradation products of anticancer drugs. For that purpose, they designed experiments, where different stress conditions led to the breakdown of parent compounds and used NMR, FAB (fast atom bombardment) or ESI (electrospray ionization) spectrometry, FT-IR, or other mass spectrometry techniques to identify degradation products. These experiments were mostly based on the clinical and biological safety of administration the medicaments and the knowledge of their possible degradation pathways in order to assess a risk of creation toxic and dangerous compounds for the clinical patients. On the other hand, Muñoz et al. (1996) presented the combined approach, where they focused on the hydrolysis of IF and various parameters regarding that process and additionally identified one main degradation product. Other authors like Shivakumar and Dwivedi (2015) provide some data on identified degradation products of CP; however, due to many differences in the experimental set up, it is hard to compare our results to those.

**Fig. 6:**
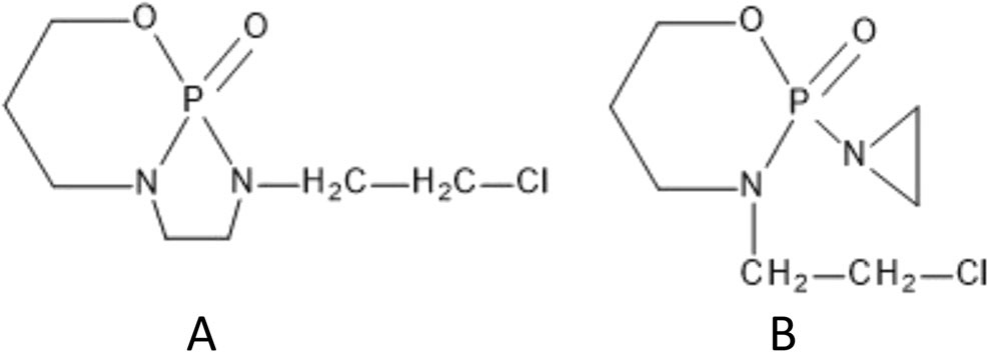
Possible structures of IF (structure A and B) and CP (structure A) degradation products proposed by (Kaijser et al. 1996) corresponding to ion m/z 225 found in our research

For these reasons, in our study, we have made some preliminary attempts to identify the potential degradation products that were produced during the breakdown of unstable compounds found in this study (CP and IF). The LC-MS system with ion trap analyzer was used for this purpose. Samples before and after the extended tests were analyzed, as well as blank and control samples. The gradient method with a slow increase of organic phase was applied to detect as much ions as possible during the TIC (total ion current) mode. The obtained results suggest that there have been some new compounds produced; nevertheless, their structure could not be determined exactly. Many of them were probably very polar and co-eluted at the beginning of the analysis. Moreover, some of them could be at a very low level of concentration; thus, the equipment was not sensitive enough to detect them. Nevertheless, by the comparison of ions detected in the control samples, blank samples, and test samples, some of the compounds were detected as potential products of hydrolytic degradation of CP and IF. The ions of *m/z* = 207 and 218 (in positive ion mode) for CP at pH 4 and 20 °C and 225 (in positive ion mode) for CP and IF at pH 9 and 70 °C were found only in the test samples (at the end of the extended test). According to the literature, there are possible products of alkaline hydrolysis of IF with a molecular mass of 224 g mol^−1^ (Kaijser et al. 1991), whose structures are presented in the Fig. 6. Thereby, *m/z* of 225 would refer to [M + H]^+^ (pseudo-molecular ion in positive ionization mode).

**Fig. 7:**
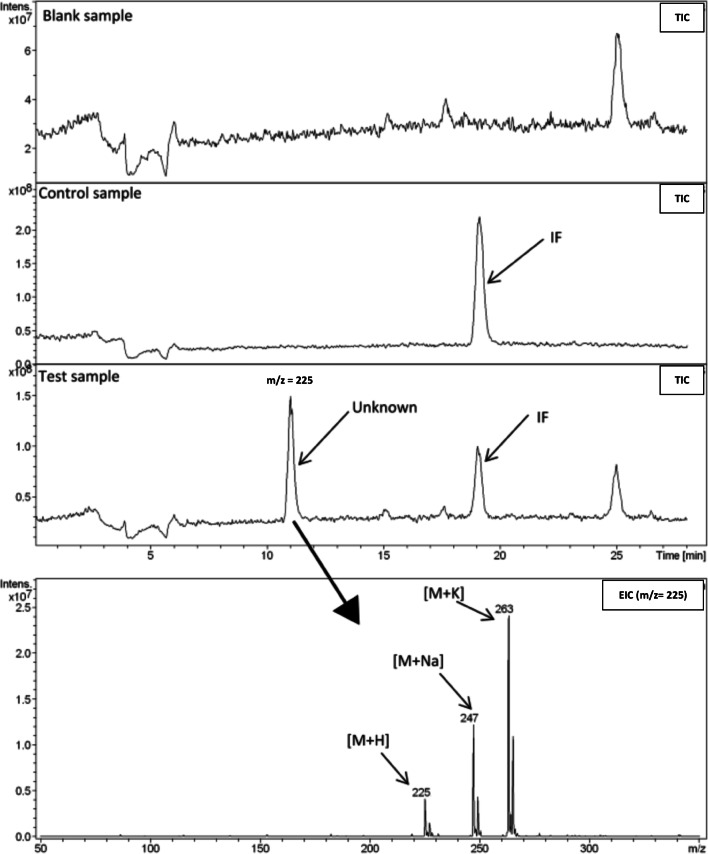
The comparison of TIC (Total Ion Current) chromatograms obtained for blank sample, control sample and test sample of IF after the extended test at 70 °C, pH 9 and EIC (extracted ion chromatogram) of m/z 225, which is a potential degradation product of IF

Kaijser et al. 1996 proposed two structures: structure A was suggested based on observed degradation product of CP in gas chromatography, described as intramolecular rearrangement (Van Den Bosch and De Vos 1980). Structure B represents aziridine structure, which is more likely to be formed, according to the authors (Kaijser et al. 1996), who also pointed out the formation of similar structures during the hydrolysis of phosphoramide mustard at higher pH values (Watson et al. 1985). Additionally, these results are in agreement with Gilard et al. (1999), who seem to confirm the hypothesis, basing on the known cyclization of haloamines into aziridines. Based on our results, it might be suspected that there is probably one molecule of chlorine left in the investigated structure, due to two specific isotopic peaks with an *m/z* difference of two, which exhibit a constant height ratio (3:1), which is distinctive for chlorine presence (Fig. 7). Moreover, similar ion was found for CP at 70 °C pH 9, so it may be suggested that structures proposed in the Fig. 6, especially compound A, which is established derivate of CP (Van Den Bosch and De Vos 1980), is the probable degradation product in the test conditions.

Considering other ions found in test samples of CP (*m/z* 207 and 218), at this stage of the research we were not able to determine their structures. The literature mentioned above (Gilard et al. 1994, 1999; Kaijser et al. 1996) provides some potential structures of transformation products; however, none of the molecular masses fit to our findings.

## Conclusions

Presented results show that most of the investigated cytostatic drugs as well as one metabolite were stable under the test conditions. According to OECD guideline, 5-FU, IMT, MTX, and 7-OH-MTX are hydrolytically stable and their half-lives are at least 1 year at 25 °C. This also means that the hydrolysis is of minor importance in terms of their potential elimination under environmental conditions. CP degraded fully in 5 days at 50 °C at all tested pH values, whereas IF was unstable mostly at pH 4, where about 50 % of initial concentration declined. Nevertheless, further tests indicated that at lower temperatures, both compounds are quite stable, with half-lives for CP around 80 days at 20 °C. IF at the same temperature degraded only at pH 4 for around 20 %, while it was completely stable at pH 7 and 9 for 30 days. The quickest degradation occurred at 70 °C, where t_½_ for CP was 2.5–2.6 h and for IF it was higher and dependent on pH (lowest at pH 4 – 11.7 h, highest for pH 7–16.9 h). At 50 °C, t_½_ for CP at three pH values were in the range 27.6–29.4; however, for IF at pH 4 it was 86.5 h, pH 7–241.9 h, and pH 9–191.7 h. Moreover, the activation energy was at similar level for CP at every tested pH value (110.5–111.2 kJ mol^−1^), whereas for IF it was 84.2 kJ mol^−1^ at pH 4, 122.2 kJ mol^−1^ at pH 7, and 120.1 kJ mol^−1^ at pH 9. It supports the data on the thesis that IF is more susceptible for hydrolysis in acidic conditions. In environmental context, general stability of cytostatics suggests that they may be persistent in the environment, being a threat to biota. Additionally, preliminary attempts were performed in order to determine the potential hydrolysis products of CP and IF; however, for this purpose, further studies are still needed.

## Supplementary Information

ESM 1 (DOCX 1.55 mb)

## Data Availability

Most of the data generated or analyzed during this study are included in this published article (and its supplementary materials); if necessary, more data are available from the corresponding author on a reasonable request.
