# Supplementary material for: Hydrolytic stability of anticancer drugs and one metabolite in the aquatic environment
Source: Environ Sci Pollut Res Int. 2021 Jun 8;28(41):57939–51. doi: 10.1007/s11356-021-14360-0 (PMC8536627; doi:10.1007/s11356-021-14360-0)
Supplement: Supplementary file 1 — (DOCX 1.55 mb) [file 11356_2021_14360_MOESM1_ESM.docx]

**Supplementary Material**

**for**

**Hydrolytic stability of anticancer drugs and one metabolite in the aquatic environment**

Michał Toński, Joanna Dołżonek, Piotr Stepnowski, Anna Białk-Bielińska*

Department of Environmental Analysis, Faculty of Chemistry, University of Gdańsk, ul. Wita Stwosza 63, 80-308 Gdańsk, Poland

**Content:**2 Tables

2 Figures

*Corresponding authors e-mail: anna.bialk-bielinska@ug.edu.pl; phone (+48 58) 5235207

**Table S1** Parameters of the HPLC-UV-Vis methods used for the quantitative analysis of the selected compounds in this study

| Compound | CP | IF | 5-FU | IMT | MTX | 7-OH-MTX |
| --- | --- | --- | --- | --- | --- | --- |
| Molar mass [g mol^-1^] | 261.09 | 261.09 | 130.08 | 493.60 | 454.44 | 470.44 |
| Detection wavelength [nm] | 200 | 200 | 266 | 265 | 302 | 302 |
| Phase A | Acetonitrile | | | | | |
| Phase B | Water | | | 0.1 % HCOOH | | |
| Composition of mobile phase (V/V) | 25:75 | 25:75 | 5:95 | 15:85 | 10:90 | 15:85 |
| Injection volume [µL] | 50 | 50 | 10 | 50 | 50 | 50 |
| Flow rate [mL min^-1^] | 0.7 | 0.7 | 0.7 | 0.7 | 1 | 1 |
| LOD  [mg L^-1^] | 0.080 | 0.080 | 0.017 | 0.17 | 0.021 | 0.042 |
| LOQ  [mg L^-1^] | 0.250 | 0.250 | 0.050 | 0.5 | 0.063 | 0.125 |
| Accuracy [%] | 76.1 – 112.9 | 81.7 – 100.2 | 86.2 – 118.3 | 91.4 – 100.2 | 87.7 – 120.6 | 93.3 – 99.7 |
| Precision [%] | 0.4 – 13.5 | 0.2 –3.1 | 0.1 – 6.5 | 0.9 – 20.6 | 1.2 – 9.1 | 0.4 – 2.6 |

**Table S2** LC-MS method parameters applied for qualitative analysis of CP and IF samples collected after the extended tests

| LC conditions | | | MS conditions | |
| --- | --- | --- | --- | --- |
| Mobile phase A | H_2_O | | **Ion source** | ESI (Ultra scan) |
| Mobile phase B | Acetonitrile | | **Scan mode** | Full scan |
| Gradient elution programme | Time [min] | %B | **Ion mode** | Positive and negative |
|  | 0  20  23  28 | 20  35  20  20 |  |  |
| Mobile phase flow  [mL min^-1^] | 0.4 | | **Monitored m/z range** | 50 – 350 |
| Injection volume  [µL] | 50 | | **Drying gas parameters** | 10 L min^-1^, 350 °C |
| Analytical wavelength [nm] | 200, 223, 254, 280 | | **Capillary voltage [V]** | 4000 |
| Column | Phenomenex Gemini C18  (5 µm, 110 Å,  4.6 x 150 mm) | | **Nebulizer pressure** | 30 PSI |


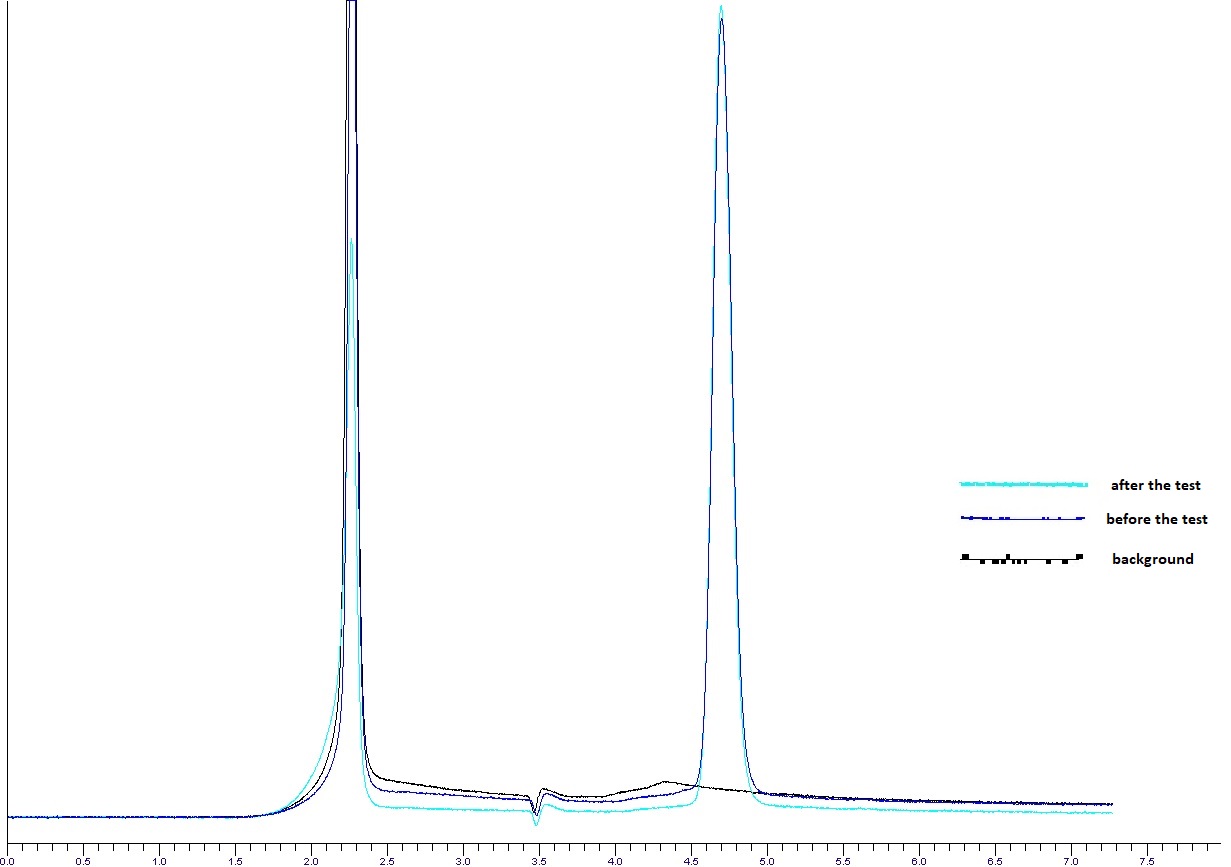


**Figure S1** Chromatograms obtained during the evaluation of hydrolytic stability of 5-FU at pH 7 before and after the preliminary test

**Figure S2** Degradation levels of CP and IF during extended tests at each pH and temperature (the error bars represent standard deviation)
